# Complete Genome Sequencing of Influenza A Viruses within Swine Farrow-to-Wean Farms Reveals the Emergence, Persistence, and Subsidence of Diverse Viral Genotypes

**DOI:** 10.1128/JVI.00745-17

**Published:** 2017-08-24

**Authors:** Andres Diaz, Douglas Marthaler, Marie Culhane, Srinand Sreevatsan, Moh Alkhamis, Montserrat Torremorell

**Affiliations:** a Veterinary Population Medicine Department, College of Veterinary Medicine, University of Minnesota, Saint Paul, Minnesota, USA; b Veterinary Diagnostic Laboratory, College of Veterinary Medicine, University of Minnesota, Saint Paul, Minnesota, USA; c Environment and Life Sciences Research Center, Kuwait Institute for Scientific Research, Kuwait City, Kuwait; St. Jude Children's Research Hospital

**Keywords:** complete genome sequencing, diseases of swine, influenza A virus diversity, influenza A virus emergence, influenza A virus epidemiology, influenza A virus persistence, influenza in swine herds, next-generation-sequencing technologies, swine influenza, swine influenza epidemiology

## Abstract

Influenza A viruses (IAVs) are endemic in swine and represent a public health risk. However, there is limited information on the genetic diversity of swine IAVs within farrow-to-wean farms, which is where most pigs are born. In this longitudinal study, we sampled 5 farrow-to-wean farms for a year and collected 4,190 individual nasal swabs from three distinct pig subpopulations. Of these, 207 (4.9%) samples tested PCR positive for IAV, and 124 IAVs were isolated. We sequenced the complete genomes of 123 IAV isolates and found 31 H1N1, 26 H1N2, 63 H3N2, and 3 mixed IAVs. Based on the IAV hemagglutinin, seven different influenza A viral groups (VGs) were identified. Most of the remaining IAV gene segments allowed us to differentiate the same VGs, although an additional viral group was identified for gene segment 3 (PA). Moreover, the codetection of more than one IAV VG was documented at different levels (farm, subpopulation, and individual pigs), highlighting the environment for potential IAV reassortment. Additionally, 3 out of 5 farms contained IAV isolates (*n* = 5) with gene segments from more than one VG, and 79% of all the IAVs sequenced contained a signature mutation (S31N) in the matrix gene that has been associated with resistance to the antiviral amantadine. Within farms, some IAVs were detected only once, while others were detected for 283 days. Our results illustrate the maintenance and subsidence of different IAVs within swine farrow-to-wean farms over time, demonstrating that pig subpopulation dynamics are important to better understand the diversity and epidemiology of swine IAVs.

**IMPORTANCE** On a global scale, swine are one of the main reservoir species for influenza A viruses (IAVs) and play a key role in the transmission of IAVs between species. Additionally, the 2009 IAV pandemics highlighted the role of pigs in the emergence of IAVs with pandemic potential. However, limited information is available regarding the diversity and distribution of swine IAVs on farrow-to-wean farms, where novel IAVs can emerge. In this study, we studied 5 swine farrow-to-wean farms for a year and characterized the genetic diversity of IAVs among three different pig subpopulations commonly housed on this type of farm. Using next-generation-sequencing technologies, we demonstrated the complex distribution and diversity of IAVs among the pig subpopulations studied. Our results demonstrated the dynamic evolution of IAVs within farrow-to-wean farms, which is crucial to improve health interventions to reduce the risk of transmission between pigs and from pigs to people.

## INTRODUCTION

In 2009, a novel influenza A virus (IAV) emerged from swine IAVs, caused the first influenza pandemic of the 21st century, and changed perceptions of the role of pigs in the ecology of IAVs between species (1, 2). IAVs are endemic in wild waterfowl (3), humans (4), and pigs (5, 6), and recent studies have demonstrated that pigs not only play a key role in the maintenance and adaptation of IAVs from avian to human transmission, but also serve as a key host for viral distribution on a global scale (7). IAVs can also infect poultry (8, 9), horses (10), cats (11), dogs (12), and some marine mammals (13), and a distant genetic lineage of IAV has been identified in bats (14, 15). Zoonotic IAVs can cause pandemic infections (16); however, not all zoonotic IAV infections exhibit sustained human-to-human transmission (17, 18). Except for the 2009 pandemic IAV, no other swine origin transmission has acquired the ability to transmit effectively between humans; however, several reverse-zoonotic events have resulted in human IAVs becoming established in swine populations (19–21).

IAVs are orthomyxoviruses with eight single-stranded, negative-sense RNA gene segments that encode at least 12 different proteins (22). IAV genome segments include those encoding polymerase B2 (PB2) (segment 1), polymerase B1 (PB1) (segment 2), polymerase A (PA) (segment 3), hemagglutinin (HA) (segment 4), nucleoprotein (NP) (segment 5), neuraminidase (NA) (segment 6), matrix (M) (segment 7), and nonstructural protein (NS) (segment 8). RNA viruses change rapidly over time (23), and IAVs are no exception. However, not all IAV gene segments change at the same rate, and substitution rates can be host species specific (24). Additionally, the segmented nature of the IAV genome allows two or more IAVs to exchange gene segments (reassort) during replication (25, 26), increasing exponentially the potential for virus diversification. IAVs are commonly found on North American pig farms (27–29), where H1N1, H1N2, and H3N2 subtypes are frequently identified (30). At the HA level, in North America, these IAVs cluster in six antigenically and phylogenetically distinct H1 clades (α, β, γ1, γ2, δ1, and δ2) (31, 32) and four H3 clusters (I, II, III, and IV) (33). The genetic diversity of IAVs in North American pigs has been associated with the geographical distribution and movement of pigs (20), and the international trade in pigs is considered a predictor pathway of genetically related IAVs being introduced from the United States and Europe into Asia (34, 35).

Currently, there are almost 70 million pigs in the United States, including 6 million breeding stock. The majority of these pigs are born on farrow-to-wean farms, although other pigs are born on farrow-to-finish farms, born wild, or even born at production sites to produce pet pigs. On farrow-to-wean farms, artificial insemination, gestation, and farrowing take place continuously (36), and pigs are weaned at about 21 days of age. After weaning, the pigs are transported to a separate site to be reared to the next production phase (nursery farms) or until they go to market (wean-to-finish farms), which happens at about 24 weeks of age (37). Moreover, farrow-to-wean farms house different subpopulations of pigs with different ages, production purposes, and susceptibilities to IAV infection (38, 39). These subpopulations include sows (mothers of piglets), replacement animals for the sows (gilts), and piglets (pigs from birth to weaning). Gilts are introduced to the farm on a regular basis and replace sows at a yearly rate of 45 to 55% (37). Moreover, suckling piglets represent approximately 40% of the resident pig population, and every week newborn piglets replace the suckling pigs that are weaned at 3 weeks of age. Therefore, swine-breeding farrow-to-wean farms house dynamic pig subpopulations in which suckling piglets represent the largest subpopulation and have the highest replacement rate among all the different pig subpopulations present.

Despite all the knowledge gained on IAV diversity in pigs as a result of the increased surveillance efforts of the last decade, limited information is available on IAV genetic diversity and evolution at the farm level. We hypothesize that the population dynamics present on farrow-to-wean farms play a key role in the introduction and maintenance of swine IAVs over time. Different pig subpopulations and their unique replacement rates may represent different ecological niches for IAV replication and evolution. Understanding the evolution and diversity of IAVs among swine subpopulations on farrow-to-wean farms is crucial to unraveling the mechanisms by which IAVs persist for prolonged periods. Therefore, we characterized the complete genomes of IAVs during infection of pigs under field conditions and demonstrated the dynamic occurrence of IAVs in pig subpopulations that are present on farrow-to-wean farms. The results from this study provide a better understanding of IAV diversity and persistence at the farm level, knowledge that is required to design more effective health interventions to control IAV infection in pigs and to reduce its zoonotic potential.

## RESULTS

**FIG 1:**
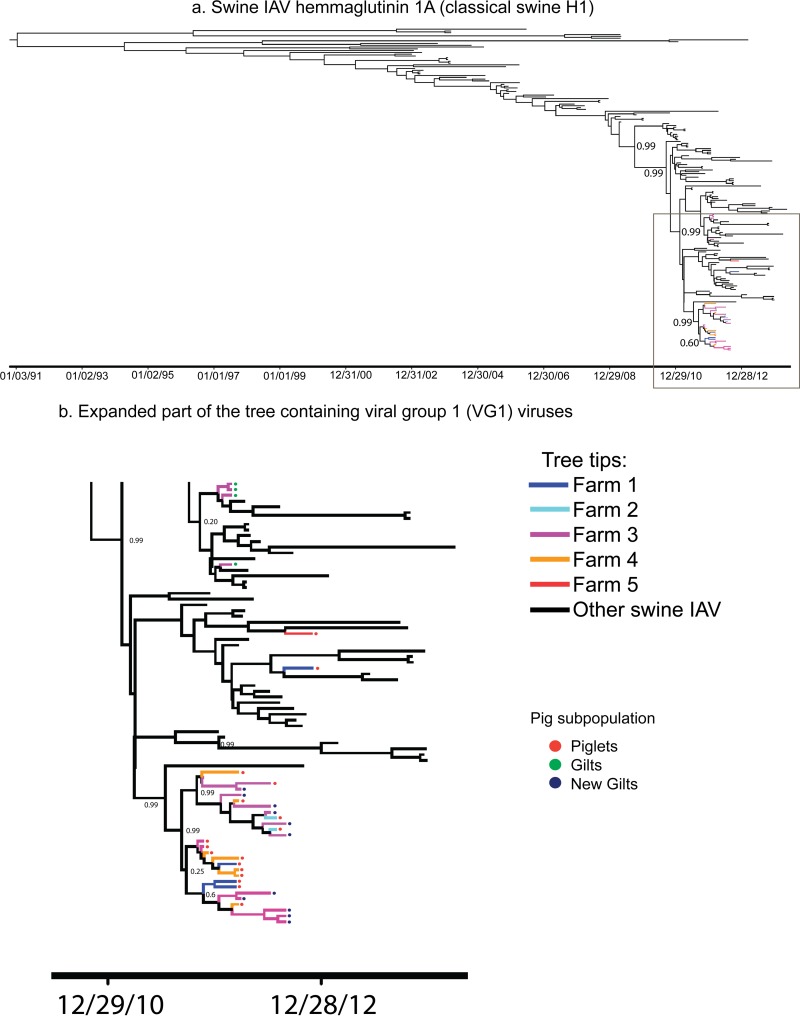
Maximum clade credibility tree of swine influenza virus H1 lineage 1A hemagglutinin illustrating the time scale distribution of VG1 by farm (1 to 5) and pig subpopulation (piglets, gilts, and new gilts). The tree is color coded based on IAVs from farms 1 to 5 or other IAVs circulating in the United States between January 2003 and October 2014, as indicated. The colored dots next to the tree tips indicate pig subpopulations. MCMC posterior probabilities are shown for key nodes.

**FIG 2:**
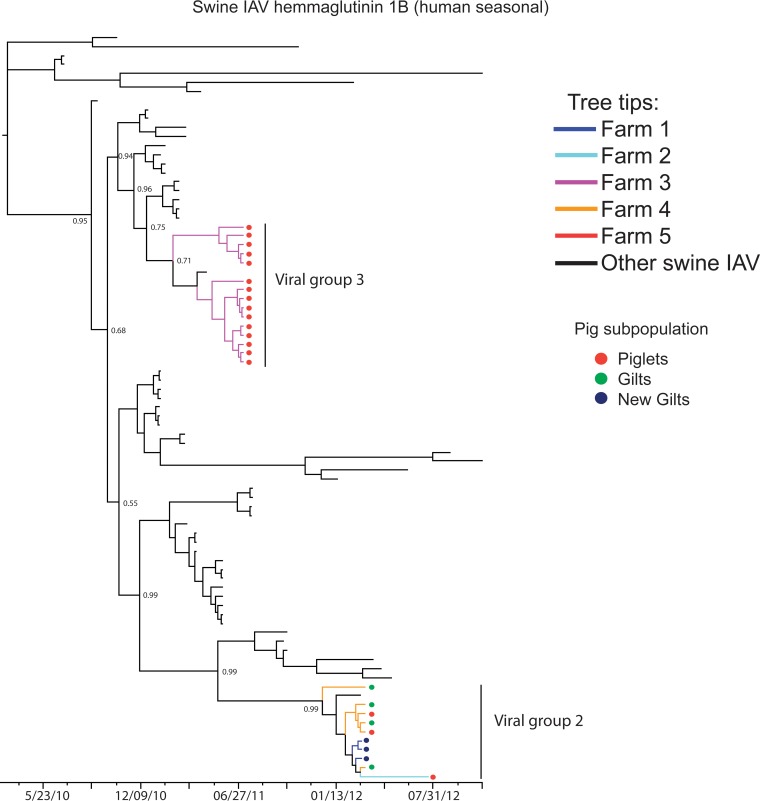
Maximum clade credibility tree of swine influenza virus H1 lineage 1B hemagglutinin illustrating the time scale distribution of VG2 and VG3 by farm (1 to 5) and pig subpopulation (piglets, gilts, and new gilts). The tree is color coded based on IAVs from farms 1 to 5 and other IAVs circulating in the United States between January 2003 and October 2014, as indicated. The colored dots next to the tree tips indicate pig subpopulations. MCMC posterior probabilities are shown for key nodes.

**FIG 3:**
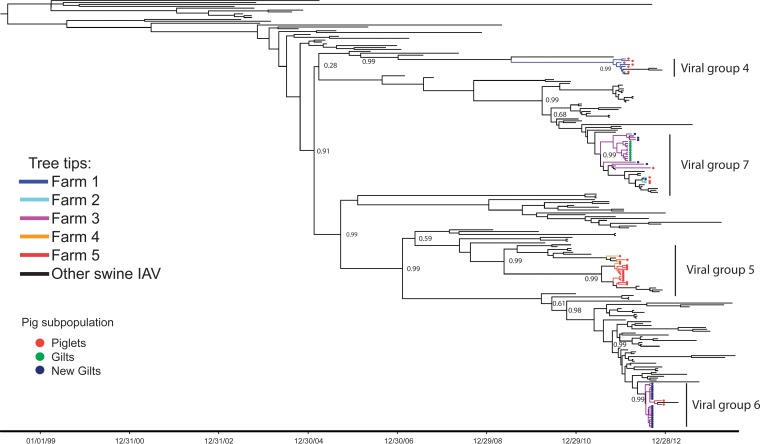
Maximum clade credibility tree of swine influenza H3 hemagglutinin illustrating the time scale distribution of VG4 to VG7 by farm (1 to 5) and pig subpopulation (piglets, gilts, and new gilts). The tree is color coded based on IAVs from farms 1 to 5 and other IAVs circulating in the United States between January 2003 and October 2014, as indicated. The colored dots next to the tree tips indicate pig subpopulations. MCMC posterior probabilities are shown for key nodes.

**FIG 4:**
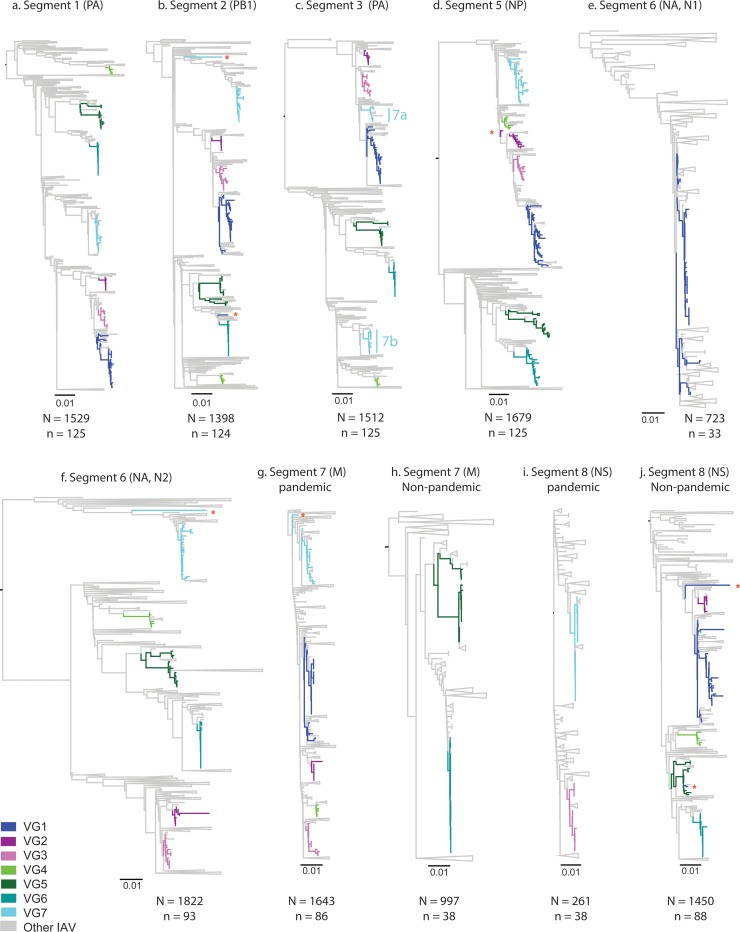
Approximately maximum-likelihood trees for IAV gene segments 1 (PA), 2 (PB1), 3 (PA), 5 (NP), 6 (NA), 7 (M), and 8 (NS). The trees include gene sequences (N) from IAVs circulating in the United States between January 2003 and October 2014. The tree branches containing sequences from this study (n) are color coded based on viral groups (VG1 to VG7). Sequences that did not cluster within the expected clade (based on their VG classification) are marked with red asterisks. In panel c, 7a and 7b illustrate the additional clades identified for viruses classified as VG7.

**FIG 5:**
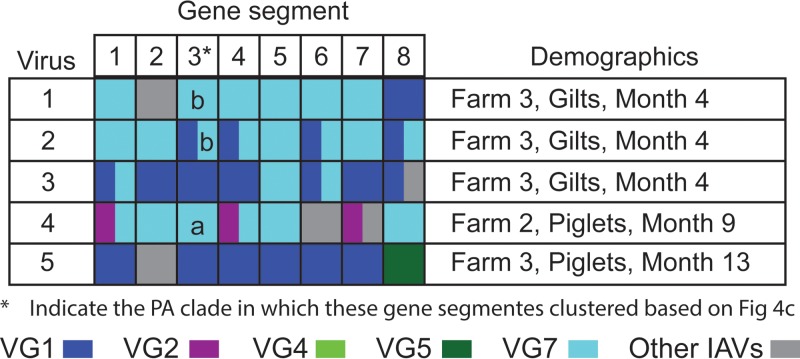
Mixed influenza A viruses. The genome constellations of IAV isolates (*n* = 5) with gene segments from two or more VGs are shown. The farm, subpopulation, and sampling month are indicated. The gene segments are color coded based on the VG assigned, as indicated. Gray indicates sequences that were closer to those of other IAVs not isolated in this study.

Five swine farrow-to-wean farms were followed for a year, and 4,190 individual nasal swabs were collected from three distinct pig subpopulations (piglets, gilts, and new gilts). Two hundred and seven samples (4.9%) tested PCR positive for IAV (cycle threshold [*C_T_*] < 35), 124 IAVs were isolated (59.9%), and the genomes of 123 IAV isolates were sequenced (Table 1). Of these 123 IAV isolates, 120 (97.5%) contained a single IAV subtype, with H1N1 (*n* = 31; 25.2%), H1N2 (*n* = 26; 21.1%), or H3N2 (*n* = 63; 51.2%) IAV. The remaining three contained mixed IAV subtypes (H1N1/H3N2, H1N1/N2, and H1N2/H3N2). For this study, these 123 IAV isolates were grouped into seven HA influenza A viral groups (VGs) named VG1 to VG7 based on their HA lineages (H1 1A, H1 1B, or H3) (33, 40), H1 clades (α, β, γ1, γ2, δ1, and δ2) (31, 32), H3 clusters (I, II, III, and IV) (33, 41), and HA pairwise sequence identity (Table 2 and Fig. 1 to 3; see Fig. S1 to S3 in the supplemental material). At the HA level, the pairwise sequence identity within VGs was higher than 98%. The remaining IAV gene segments were classified based on the 7 HA VGs created for this study, and all, excluding gene segment 3, also clustered into 7 clades (Fig. 4) (Gamma20-based likelihood [see Materials and Methods] > 0.85). Eight clades were identified for segment 3 (PA) (Fig. 4c), in which clade 7 was broken into clades 7a (Gamma20-based likelihood = 0.99) and 7b (Gamma20-based likelihood = 0.95). Additionally, viruses within VG1, -2, -3, -4, and -7 had matrix genes (segment 7) of pandemic origin (Fig. 4g), and VG3 and -7 had nonstructural genes (segment 8) of pandemic origin (Fig. 4i). Furthermore, we identified 5 IAVs containing viral gene segments from more than one VG or gene segments that were closer to those of other IAVs circulating in the United States than to those of the IAV isolated during this study (Fig. 5).

**TABLE 1:** Total numbers of nasal swabs collected; samples positive for IAV by RRT-PCR used for virus isolation; and IAV isolates distributed by farm, subpopulation, and subtype

Farm	Subpopulation	Total no. of swabs collected	No. of IAV RRT-PCR-positive samples (%)	No. of IAV isolates (%)^*a*^	IAV subtypes^*a*^
1	New gilts	119	1 (0.8)	0 (0)	NA
	Gilts	360	0 (0)	NA^*b*^	NA
	Piglets	358	21 (5.9)	12 (57)	H1N1 (*n* = 4), H3N2 (*n* = 7)
	Subtotal	837	22 (2.6)	12 (54)	H1N1 (*n* = 4), H3N2 (*n* = 7)
2	New gilts	119	0 (0)	NA	NA
	Gilts	360	0 (0)	NA	NA
	Piglets	358	7 (2.0)	5 (71)	H1N1 (*n* = 2), H1N2 (*n* = 2), H1N2/H3N2 (*n* = 1)
	Subtotal	837	7 (0.8)	5 (71)	H1N1 (*n* = 2), H1N2 (*n* = 2), H1N2/H3N2 (*n* = 1)
3	New gilts	268	72 (26.9)	40 (55)	H1N1 (*n* = 12), H3N2 (*n* = 28)
	Gilts	359	19 (5.3)	14 (73)	H1N1 (*n* = 2), H3N2 (*n* = 10), H1N1/H3N2 (*n* = 1), H1N1/N2 (*n* = 1)
	Piglets	360	20 (5.6)	20 (100)	H1N1 (*n* = 3), H1N2 (*n* = 15), H3N2 (*n* = 2)
	Subtotal	987	111 (11.2)	74 (66)	H1N1 (*n* = 17), H1N2 (*n* = 15), H3N2 (*n* = 40), H1N1/H3N2 (*n* = 1), H1N1/N2 (*n* = 1)
4	New gilts	60	0 (0)	NA	NA
	Gilts	349	19 (5.4)	4 (21)	H1N2 (*n* = 4)
	Piglets	360	21 (5.8)	15 (71)	H1N1 (*n* = 7), H1N2 (*n* = 5), H3N2 (*n* = 3)
	Subtotal	769	40 (5.2)	19 (48)	H1N1 (*n* = 7), H1N2 (*n* = 9), H3N2 (*n* = 3)
5	New gilts	60	0 (0)	NA	NA
	Gilts	340	0 (0)	NA	NA
	Piglets	360	27 (7.5)	14 (52)	H1N1 (*n* = 1), H3N2 (*n* = 13)
	Subtotal	760	27 (3.6)	14 (52)	H1N1 (*n* = 1), H3N2 (*n* = 13)
Total		4,190	207 (4.9)	124 (60)	H1N1 (*n* = 31), H1N2 (*n* = 26), H3N2 (*n* = 63), H1N1/H3N2 (*n* = 1), H1N1/N2 (*n* = 1), H1N2/H3N2 (*n* = 1)

a One IAV isolate from the piglets on farm 1 was not successfully sequenced.

b NA, not applicable.

**TABLE 2:** Influenza A viruses isolated during the study period distributed by viral group (VG1 to VG7), subtype, and percent hemagglutinin sequence identity (ClustalX) within and between groups

Virus group	No. of sequences	HA and NA subtype	HA North American clade or cluster^*a*^	HA global clade^*a*^	% sequence identity with:						
					VG1	VG2	VG3	VG4	VG5	VG6	VG7
VG1	33	H1N1	Gamma	1A.3.3.3	98.3–100	74.7–75.2	75.0–75.0	48.8–49.2	48.7–49.5	48.4–48.8	48.8–49.2
VG2	10	H1N2	Delta 1	1B.2.2.2	74.7–75.2	99.9–100	97.9–98.1	50.4–50.5	50.6–50.7	50.4–50.6	49.9–50.2
VG3	15	H1N2	Delta 1	1B.2.2.2	75.0–75.0	97.9–98.1	99.6–100	50.2–50.4	50.4–50.8	50.4–50.7	50.0–50.4
VG4	6	H3N2	Cluster IV	NA	48.8–49.2	50.4–50.5	50.2–50.4	99.7–100	94.2–94.8	93.4–93.7	93.1–93.8
VG5	15	H3N2	Cluster IV	NA	48.7–49.5	50.6–50.7	50.4–50.8	94.2–94.8	98–100	95.1–95.6	93.9–94.6
VG6	23	H3N2	Cluster IV	NA	48.4–48.8	50.4–50.6	50.4–50.7	93.4–93.7	95.1–95.6	99.9–100	93.0–93.4
VG7	23	H3N2	Cluster IV	NA	48.8–49.2	49.9–50.2	50.0–50.4	93.1–93.8	93.9–94.6	93.0–93.4	99.1–100

a H1 North American clade and global clade classifications were established using the Swine H1 clade classification tool from the Influenza Research Database (http://www.fludb.org), and H3 cluster classification was estimated based on previous studies. NA, not applicable.

**FIG 6:**
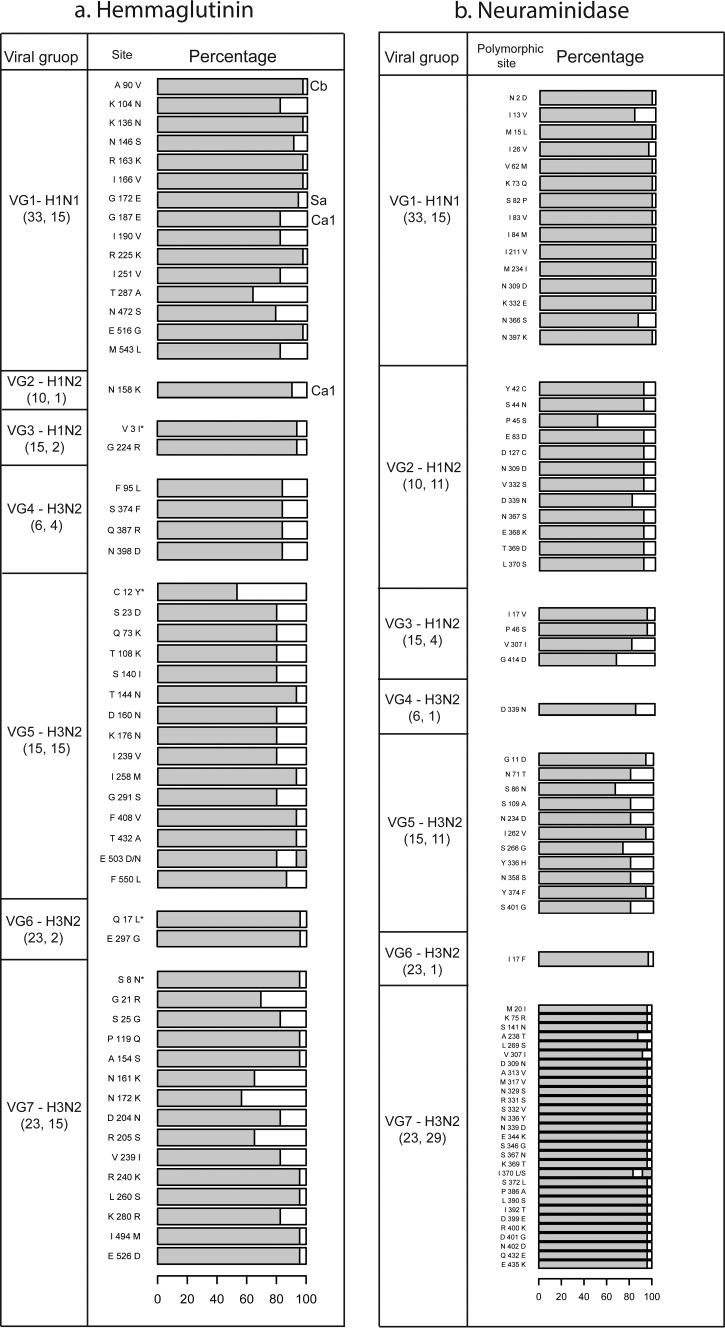
Polymorphic amino acid sites within the hypothetical hemagglutinins and neuraminidases of IAV isolates. Viral groups and IAV subtypes are indicated. The numbers in parentheses are the total numbers of sequences compared and the numbers of polymorphic amino acid sites found. The polymorphic site indicates the amino acid most frequently found (first letter), the position (number), and the variant amino acid (second letter). Amino acid position numbers correspond to the entire HA or NA protein. The horizontal bars represent the percentage distribution of each amino acid at each polymorphic site based on the number of sequences compared. The asterisks indicate polymorphic sites within the signal peptide of the HA. Polymorphic sites within known H1 antigenic regions (Cb, Sa, and Ca1) are indicated.

To expand our understanding of the HA and NA diversity among the VGs identified, we translated the HA and NA nucleotide sequences into hypothetical protein sequences, and their polymorphic sites within VGs are illustrated in Fig. 6. Complete HA numbering was used (including the signal peptide). The number of polymorphic amino acid sites among HA proteins ranged between 1 and 15 (Fig. 6a). Polymorphic amino acids were lacking within the HA2 region of HA, and only four polymorphic sites were found within the signal peptide regions of four VGs (VG3, VG5, VG6, and VG7). Only a single site in HA proteins had 3 polymorphic amino acids at this position (VG5; E503D/N), while all the other sites had only 2 polymorphic amino acids. Furthermore, four polymorphic sites were within known antigenic regions of H1 hemagglutinin (90 [Cb], 159 [Ca1], 172 [Sa], and 187 [Ca1]) (42). Polymorphic sites were lacking in the six amino acid positions (145, 155, 156, 158, 159, and 189) recently identified as key determinants of swine H3 antigenicity (43). The number of polymorphic sites among NAs ranged between 1 and 29 (Fig. 6b), and a single polymorphic site had 3 polymorphic amino acids (VG7; I370L/S). Additionally, annotation of the matrix genes (segment 7) using the NCBI Flu Annotation Web service (FLAN) (44) identified a signature mutation (S31N) associated with resistance to the antiviral amantadine in 79% (*n* = 98) of the sequences, which was found in matrix genes with and without pandemic origins.

**FIG 7:**
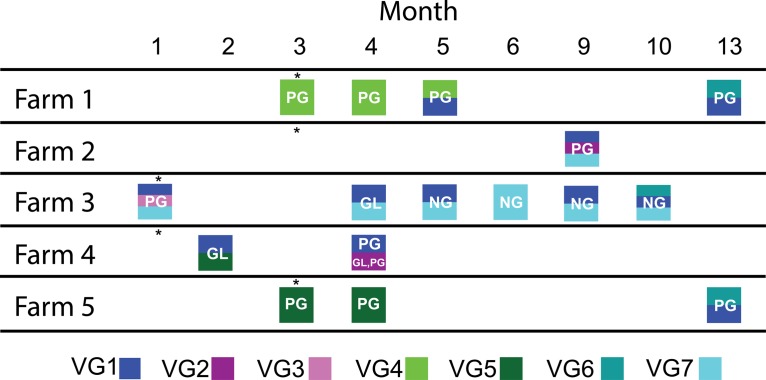
IAV isolates distributed by month, viral group, farm, and subpopulation (new gilts [NG], gilts [GL], and piglets [PG]). Month 1 corresponds to November 2011, and month 14 corresponds to December 2012 (only months in which IAVs were isolated are indicated). The colored boxes indicate the viral groups and are labeled based on the pig subpopulation from which IAVs were isolated. The asterisks indicate the month in which the sampling events started on each farm.

At the farm level, the IAV emergence (first isolation of an IAV), persistence (isolation of the same IAV over time), and subsidence (no further isolation of an IAV previously recovered) are illustrated in Fig. 7. While VG1 viruses were found on all the farms, VG3 and VG4 were found only on farm 3 and farm 1, respectively. IAVs from VG2, -3, and -6 were recovered only once on the same farm during the sampling period, while IAVs from VG1, -4, -5, and -7 were isolated multiple times. IAVs from VG1 persisted on farms 1, 3, and 4, while IAVs from VG4, -5, and -7 persisted only on farms 1, 5, and 3, respectively. Additionally, IAVs from more than one VG were found in piglets (farms 1 to 5), gilts (farm 3), and new gilts (farm 3). Furthermore, 3 different VGs were isolated from piglets and new gilts from farm 3 (months 1 and 10, respectively) and in piglets from farm 2 at month 9. Moreover, VG5 persisted for 35 days on farm 5, while VG1 and VG7 persisted simultaneously for 283 days on farm 3. Initially, VG1, -3, and -7 were cocirculating in piglets on farm 3 (month 1); then, VG1 and -7 were recovered from gilts (month 4) and new gilts (months 5, 9, and 10).

## DISCUSSION

To evaluate the genetic and antigenic diversity of IAVs in Midwest swine farrow-to-wean farms in the United States, we conducted a 1-year longitudinal study with multiple cross-sectional sampling events and characterized the complete genomes of 123 viruses isolated from three pig subpopulations. Our results demonstrated the dynamic nature of IAVs during natural infection in swine and highlighted the key role of pig subpopulations in the diversity and distribution of swine IAVs on the farms studied. At the farm level, we illustrated the emergence, persistence, and subsidence of different VGs over time and demonstrated the ongoing exposure of pig subpopulations to IAVs that were closely related to each other or clearly distinct. Moreover, the cocirculation of IAVs with different phylogenetic origins demonstrated the potential environment for viral reassortment and illustrated the power of next-generation sequencing (NGS) to differentiate IAV infections. Our findings provide further knowledge to prevent and control swine IAV infections in breeding herds, taking into account the plasticity of the IAV genome and the dynamic nature of pig subpopulations in the contemporary swine industry.

Understanding IAV evolution in endemically infected swine populations is complex due to both the molecular characteristics of the virus and the dynamic replacement of hosts in the contemporary swine industry. In our study, multiple VGs coexisted at the farm level in one or multiple pig subpopulations. However, some VGs persisted over time while others appeared to “subside” or disappear. The persistence and cocirculation of swine IAVs on pig farms has been reported previously (30) and facilitates antigenic drift and shift over time. Nevertheless, the extent of the analysis performed in this study provided information on the epidemiology and molecular diversity of swine IAVs on farrow-to-wean farms at a level that has not been previously documented.

The ecological circumstances that allow IAVs to persist or disappear in swine populations are still not clearly determined. The simplest explanation for this dynamic occurrence in swine populations is the antigenic diversity of IAVs. At the HA level, swine IAVs with different genetic lineages are known to have different antigenic properties (31, 43, 45), and in this study, we recovered IAVs with known antigenic differences (H1 1A versus H1 1B versus H3 viruses). Moreover, nucleotide and amino acid mutations can also happen rapidly after infection of pigs with or without maternally derived immunity (46). In this study, we found polymorphic amino acid sites within the HAs of both H1 and H3 viruses, and the piglets sampled were expected to have diverse maternally derived immunity to IAVs, given the distribution of swine IAVs in the United States (31, 41). Whether polymorphic amino acids appeared due to maternally derived immunity or other viral mechanisms on farrow-to-wean farms is not known. Hence, the effect of maternally derived immunity on the epidemiology and diversity of swine influenza should be further investigated.

IAVs are transmitted with a collection of HA alleles (sequence variants) that can emerge or disappear during infection of humans and pigs (47–49). This dynamic transmission of different IAV alleles during infection of vaccinated pigs involves the entire genome of the virus, not only HA (49), and suggests that the emergence, maintenance, and subsidence of diverse IAV genotypes on farrow-to-wean farms may be due to the combination of different alleles present at the individual level. Moreover, the cocirculation of two or more IAVs contributes to genetic reassortment and the emergence of novel IAVs (50, 51). In North America, the genetic diversity of swine IAVs has increased dramatically since the emergence of the triple-reassortant internal gene (TRIG) cassette in 1998 (5, 41) and the introduction of several human IAVs into swine populations, including the 2009 pandemic virus (19, 21). In this study, we recovered several IAVs with genome constellations that suggested IAV reassortment events. These genome constellations included gene segments of VGs isolated during this study and gene segments closer to IAVs not recovered during our study. Whether these reassortant viruses emerged within the farms studied, on the farms that supplied the gilts, or on other pig farms is not clear. However, the cocirculation of different IAVs and the isolation of IAVs with mixed genotypes indicate the conditions for reassortment were present during the study period. In contrast, the identification of gene segments that were more closely related to those of IAVs currently circulating in the United States than to those of IAVs identified in this study suggests external sources (e.g., other pigs) are also important for the emergence of reassortant viruses on farrow-to-wean farms. Therefore, interventions to control IAV infection on swine farrow-to-wean farms should not only target transmission within herds, but also minimize the risk of new IAV introductions.

The ability of IAVs to exchange gene segments over time not only increases the mechanisms of virus diversification, but also may allow genetic traits (e.g., signature mutations) to move between IAVs. Seventy-nine percent of the IAVs isolated in our study contained a signature mutation (S31N) in the matrix gene (segment 7) that might confer resistance to amantadine (52). This high prevalence of amantadine resistance is in agreement with two previous studies of swine IAVs (53, 54). Interestingly, amantadine is not labeled for use in pigs by the U.S. Food and Drug Administration (FDA). Moreover, the incidence of human IAVs resistant to amantadine changed from 0.4% in 1994 (55) to 15.5% in 2006 (56), which could be associated with antiviral use in humans. Whether the high frequency of S31N in the matrix gene among swine IAVs is due to random events, as indicated by Baranovich et al. (53), or to reassortment events, as indicated by Krumbholz et al. (54), is not clear and must be further investigated. Nevertheless, multiple introductions of human IAVs into swine populations have led to the establishment of certain IAV gene lineages (19), and we speculate that in that process, genetic signatures of resistance to antivirals, such as amantadine, have also been incorporated. Hence, these introductions and the establishment of “foreign” IAV gene segments into swine populations could likely result in unique, for example, drug-resistant, genotypes.

Moreover, IAV infection has been associated with pig subpopulations (38) and the age of pigs (39). In the United States, there are almost 70 million commercial pigs, including approximately 6 million sows. Annually, gilts replace approximately 50% (45 to 55%) of the sow population (37). Additionally, sows can farrow 2.3 times a year and wean around 10 piglets after each farrowing event. Therefore, in a 1,000-sow herd, ∼23,000 piglets are born per year (∼442 pigs every week) and ∼500 new gilts are introduced every year (∼10 gilts per week), illustrating the high replacement rate in pig subpopulations. Hence, we speculate that pig population dynamics within farrow-to-wean farms is a significant factor associated with IAV diversity, the introduction of novel IAVs into swine farrow-to-wean farms, and the emergence of reassortant IAVs.

We recognize that our results do not represent the overall dynamics of swine IAV infections on farrow-to-wean-farms in the Midwestern United States, given our herd selection bias. Additionally, we could have missed some IAVs over time, given our study design, or induced selection by isolating IAV rather than sequencing IAV directly from the nasal swabs. Furthermore, our sample size did not allow us to explore the association between the genetic diversity of IAVs and pig subpopulations. Moreover, we could not identify the source or directionality of IAV transmission on these farms. Since new gilts are introduced into the farms from external sources, we speculate that new gilts are the most likely source of the introduction of new IAVs into farrow-to-wean farms, although air (57), fomites (58), and humans (19–21) can also represent risks for new IAV infections in pigs. Alternatively, if piglets are the reservoir of IAVs at the farm level, new gilts could become infected with resident viruses every time after arrival, allowing the amplification and diversification of IAVs within a naive subpopulation. Future studies sampling gilts at arrival could clarify the relative importance of gilts as a source of new IAVs for farrow-to-wean farms versus their role in amplifying resident IAVs.

In conclusion, our study demonstrated the complex and dynamic diversity of swine IAVs during infection of pigs on farrow-to-wean farms. Complete genome amplification and NGS technologies allowed us to characterize with more precision the complete genomes of IAVs over time. We showed that IAVs can be sustained for prolonged periods and that distinct IAVs can coexist within and between subpopulations on these farms. Thus, pigs on farrow-to-wean farms are repeatedly exposed to IAVs that are closely related to each other or clearly distinct. Our results also indicated that pig population dynamics, along with the viral mechanisms of genetic diversification, should be taken into account in elucidating the diversity and evolution of swine IAVs. We speculate that if transmission of IAVs is reduced on farrow-to-wean farms, then the distribution of IAVs to other pig sites after weaning will be minimized. Understanding the epidemiology and evolution of swine IAVs on farrow-to-wean farms will allow us to design more effective strategies to reduce the impact of IAV infections on swine health and to minimize the risk to public health.

## MATERIALS AND METHODS

The protocols and procedures followed throughout the study were approved by the University of Minnesota Institutional Animal Care and Use Committee (IACUC 1207B17281) and the Institutional Biosafety Committee (IBC 1208H18341). The University of Minnesota IACUC adheres to the Animal Welfare Act as Amended (7 USC 2131-2156) administered by the U.S. Department of Agriculture (USDA).

### Study design, IAV detection, and virus isolation.

A 1-year-long longitudinal study was designed with multiple cross-sectional sampling events to characterize the genetic diversity of IAVs among three different pig subpopulations on five commercial farrow-to-wean farms (farms 1 to 5) located in the Midwestern United States. All the farms were selected based on willingness to participate, had a history of IAV infection, and were sampled on a monthly basis for 12 months. While sampling events started in November 2011 for farms 3 and 4, sample collection at farms 1, 2, and 5 started in January 2012. At the farm level, we evaluated viral emergence, persistence, and subsidence.

During each visit, 30 nasal swabs (BBL CultureSwab; Becton Dickinson and Company) were collected from three pig subpopulations: (i) new gilts (replacement breeding stock on a farm for less than 4 weeks), (ii) gilts (replacement breeding stock on a farm for more than 4 weeks), and (iii) piglets (3-week-old suckling pigs). Sows were not sampled because previous studies had found that recovering IAVs from sows is frequently unsuccessful (27–29). The sample size (*n* = 30) was calculated to be 95% confident at the subpopulation level of detecting at least 1 positive sample if the prevalence was 10% or higher. New gilts were sampled during only 21 visits due to varying schedules of the delivery of replacement animals. Once collected, swabs were refrigerated and transported to the laboratory on the manufacturer's transport medium and then placed into 1.8 ml sample storage medium (Dulbecco's modified Eagle medium [DMEM], 2% bovine serum albumin [BSA] fraction V 7.5% solution [Gibco, Life Technologies], and 5% antibiotic-antimycotic [Gibco, Life Technologies] containing 10,000 IU/ml of penicillin, 10,000 μg/ml of streptomycin, and 25 μg/ml of amphotericin B [Fungizone]). Swabs in the sample storage medium were vortexed for 10 s and then stored at −80°C until IAV testing was performed.

Samples were initially tested for IAV in pools of three by reverse transcriptase real-time (RRT) PCR targeting the matrix gene, using methods described previously (59, 60). Each pool contained only samples from the same farm, month, and subpopulation. If a pool tested positive, then aliquots of the original samples were tested individually. A test was considered positive when the RRT-PCR *C_T_* value was lower than 40, and IAV isolation was attempted from all swabs with a *C_T_* value of <35. Madin-Darby canine kidney (MDCK) epithelial cells were used for IAV isolation (61). Briefly, one six-well plate (Corning, Sigma-Aldrich) was used per sample to avoid cross-contamination between samples, and two negative controls were used per plate. When the cell monolayer was ∼90% confluent, the cell growth medium was discharged, and then each well was washed twice with Hanks' solution (Gibco, Life Technologies) containing 0.15% 1-mg/ml l-1-tosylamide-2-phenylethyl chloromethyl ketone (TPCK) trypsin (Sigma-Aldrich). Two hundred microliters of 1:1 and 1:2 dilutions of the sample was used in replicates to infect 4 wells of each plate, and the two negative controls were mock infected with 200 μl of DMEM (Gibco, Life Technologies). The plates were placed into a 5% CO_2_ incubator for an hour, and then 2 ml of viral growth medium was added to each well. The viral growth medium contained DMEM (Gibco, Life Technologies), 4% BSA fraction V 7.5% solution (Gibco, Life Technologies), 0.15% 1-mg/ml TPCK trypsin (Sigma-Aldrich), and 1% antibiotic-antimycotic (Gibco, Life Technologies). The plates were observed daily and harvested if IAV cytopathic effect (CPE) was visually confirmed. If no CPE was present, the wells were harvested at 7 days postinfection for a blind passage in a new MDCK plate. A hemagglutination assay was performed on all the wells harvested. If a well lacked CPE but was hemagglutination positive, a blind passage was performed on a new MDCK plate. IAV isolation was confirmed by CPE and antigen detection using a swine influenza virus type A antigen test kit (FluDetect; Zoetis). Initial IAV-positive isolates (passage 1) were expanded into a T25 flask for adherent cells (passage 2), and these second passages were used for complete genome amplification and sequencing.

### Complete genome amplification and sequencing.

The complete genome of IAV was amplified in a single reaction as previously described (62). IAV RNA was extracted from positive isolates using a MagMax viral RNA isolation kit (Ambion, Life Technologies). RRT-PCR was performed using the SuperScript III One-Step RT-PCR system with Platinum *Taq* DNA polymerase (Invitrogen, Life Technologies). A 50-μl PCR mixture containing 10 μl DNase/RNase-free distilled water (Gibco), 25 μl 2× reaction mixture, 1 μl SuperScript III RT mixture, 1 μl (10 μM) of each primer [MBtuni12(M), ACGCGTGATCAGCAAAAGCAGG, and MBtuni13, ACGCGTGATCAGTAGAAACAAGG], and 12 μl of RNA template was prepared. PCR products were verified by gel electrophoresis, purified using a QIAquick spin kit (Qiagen), eluted in 20 ml DNase/RNase-free distilled water (Gibco, Life Technologies), and submitted for NGS using the Illumina MiSeq system (Illumina) at the University of Minnesota Genomics Center (UMGC).

The sequencing data were analyzed through the resources available at the University of Minnesota Supercomputing Institute (MSI). The sequence quality was first verified using FastQC (63), and then the sequences were trimmed using the paired-end mode of Trimmomatic (64). Sequence assembly was performed using Bowtie2 (65) and SAMTools (66) on a reference template containing 6 IAV internal gene segments (PB2 [CY099076.1], PB1 [CY099309.1], PA [CY045233.1], NP [CY009919.1], M [DQ150436.1], and NS [CY050162.1]) and 4 antigenic gene segments (H1 [FJ789832.1], H3 [KC992248.1], N1 [GU236519.1], and N2 [KC866483.1]). Consensus sequences for each contig assembled were trimmed to coding IAV gene regions, and their functionality was verified using FLAN (44).

### Phylogenetic origins and IAV diversity within and between farms.

All IAV isolates from this study were first subtyped based on their HA and NA combinations. Then, ClustalX (67) was used to estimate the HA pairwise percent identity. Additionally, H1 sequences were classified using the swine H1 clade classification tool available at the Influenza Research Database (http://www.fludb.org) (68), and H3 sequences were classified based on the swine H3 cluster I to IV classification (33, 41). Furthermore, all gene sequences recovered during the study period (*n* = 1,000) were compared to 14,401 reference sequences from swine IAVs circulating in the United States. Reference IAV sequences from the United States between 1 January 2003 and 16 October 2014 were obtained by downloading all the IAV gene sequences available from the Influenza Research Database (IRD) (68) on 16 October 2014. An additional data set from viruses recovered within the same time frame by the USDA National Veterinary Service Laboratories (NVSL) was also included. Duplicate sequences, laboratory strains, and sequences lacking a collection date (month/day/year) were excluded.

Each IAV gene segment data set (1 to 8) was initially aligned using Multiple Sequence Comparison by Log-Expectation (MUSCLE) (69). For lineage assignment, neighbor-joining methods were used to construct initial phylogenetic trees. Then, a total of 13 IAV gene segment data sets (PB2, PB1, PA, H1 1A [classical swine], H1 1B [human seasonal], H3, NP, N1, N2, pandemic M, nonpandemic M, pandemic NS, and nonpandemic NS) were used to construct approximately maximum-likelihood trees using FastTree2 (70). Pandemic and nonpandemic denominations for M and NS genes were used based on the 2009 pandemic IAV genome (1, 16). All the gene segments were found to be free from homologous-recombination events using the Recombination Detection Program version 3 (71). The best-fitting nucleotide substitution model and partitioning scheme for each gene segment were selected using the Bayesian information criterion implemented in PartitionFinder v. 1.1 (72). Subsequently, approximately maximum-likelihood trees were constructed incorporating a generalized time reversible (GTR) substitution model (70). To assess the robustness of each node within trees, local support values were estimated using the Shimodaira-Hasegawa test (73) under the discrete gamma model with 20 rate categories (Gamma20-based likelihood). Furthermore, each IAV genome constellation was established, and possible IAV reassortment events were evaluated.

To understand the viral distribution over time on farms, a time scale phylogenesis analysis was performed for HA sequences using the Markov chain Monte Carlo (MCMC) methods available in the BEAST (74) package v1.8.4. A subset of representative H1 1A (*n* = 148), H1 1B (*n* = 83), and H3 (*n* = 169) sequences circulating in the United States between January 2003 and October 2014 was selected as background data for this analysis. Time was modeled in days, and 1 January 2003 was set as day 1. Hence, evolutionary rates were estimated per day, and day estimates obtained from the model referred to the number of days before or after 1 January 2003. A relaxed uncorrelated lognormal (UCLN) molecular clock branch rate prior (75), an exponential population growth coalescent node-age prior, and a mixed GTR model of nucleotide substitution with a gamma-distributed rate variation among sites were assumed for all gene segments. The MCMC simulation was run twice for each data set, for at least 70 million iterations each, and subsampled every 1,000 iterations. For each gene segment, two replicate MCMC simulations were carried out to ensure the stability of the simulation performance. The BEAGLE library was used to improve computational performance (76). Parameter convergence was assessed using Tracer v.1.6 (77), and a minimum effective sample size (ESS) of 200 was obtained. The statistical uncertainty was estimated through the 95% highest posterior density (HPD), and the initial 10% of the chain was removed as burn-in. Runs were combined using LogCombiner v1.8.4, maximum clade credibility (MCC) trees were summarized using TreeAnnotator v1.8.4, and FigTree v1.4.3 (78) was used to annotate the final trees.

Hypothetical HAs and NAs were translated and aligned using ClustalX (67) (matrix Blosum62). Polymorphic amino acids were inferred by stripping the polymorphic sites to estimate the frequency of each amino acid and mapped to known antigenic sites of the HA (42, 43). Complete HA numbering, including the signal peptide, was used.

### Accession number(s).

The genome sequences for 123 swine influenza A viruses isolated during this study are available in GenBank under accession numbers MF194023 to MF194502.

## Supplementary Material

Supplemental material
