# Supplementary material for: Complete Genome Sequencing of Influenza A Viruses within Swine Farrow-to-Wean Farms Reveals the Emergence, Persistence, and Subsidence of Diverse Viral Genotypes
Source: J Virol. 2017 Aug 24;91(18):e00745-17. doi: 10.1128/JVI.00745-17 (PMC5571239; doi:10.1128/JVI.00745-17)
Supplement: Supplemental material [file supp_91_18_e00745-17__index.html]

Supplemental material 

# Complete Genome Sequencing of Influenza A Viruses within Swine Farrow-to-Wean Farms Reveals the Emergence, Persistence, and Subsidence of Diverse Viral Genotypes

## Supplemental material

- Supplemental file 1 -

  Fig. S1 (Approximately maximum likelihood tree for swine IAV hemagglutinin H1 lineage 1A (or classical swine IAV) circulating in the U.S. between January 2003 and October 2014.)

  Fig. S2 (Approximately maximum likelihood tree for swine IAV hemagglutinin H1 lineage 1B (or human seasonal IAV) circulating in the U.S. between January 2003 and October 2014.)

  Fig. S3 (Approximately maximum likelihood tree for swine IAV hemagglutinin H3 circulating in the U.S. between January 2003 and October 2014. )

  PDF, 1.4M
