# Supplementary material for: Complete Genome Sequencing of Influenza A Viruses within Swine Farrow-to-Wean Farms Reveals the Emergence, Persistence, and Subsidence of Diverse Viral Genotypes
Source: J Virol. 2017 Aug 24;91(18):e00745-17. doi: 10.1128/JVI.00745-17 (PMC5571239; doi:10.1128/JVI.00745-17)

Supplemental material 1. Approximately maximum-likelihood tree for swine IAV hemagglutinin H1 lineage 1A (or classical swine) circulating in the USA between January 2003 and October 2014. 1042 sequences were included for this analysis. 33 sequences correspond to the sequences recovered during this study (VG1) and are color-coded by farm 1 (blue), 2 (aqua), 3 (magenta), 4 (orange), and 5 (red). Local support values for each node under the discrete gamma model with 20 rate categories (Gamma20-based likelihood) are illustrated based on the background color of the tree.

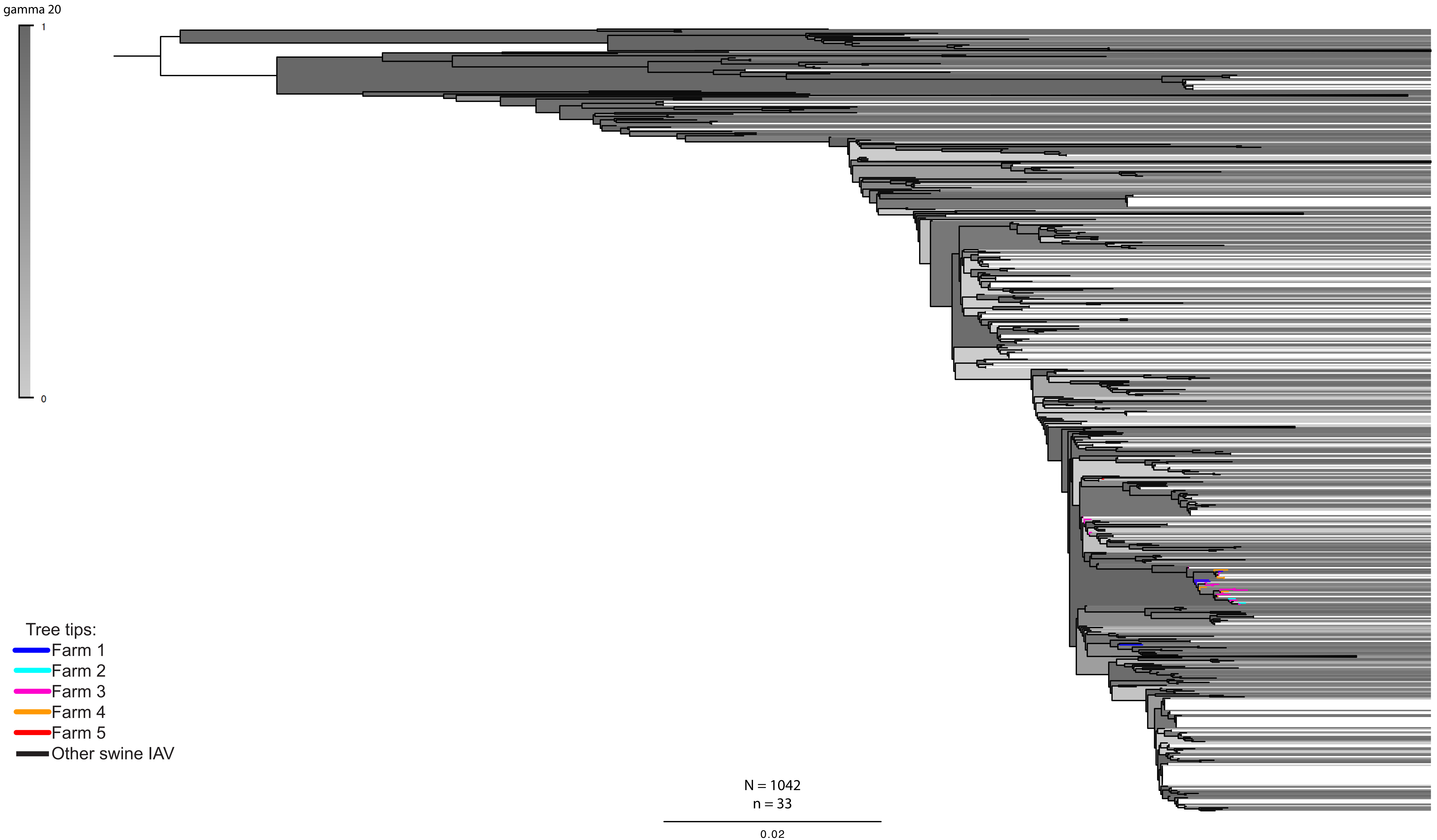

Supplemental material 2. Approximately maximum-likelihood tree for swine IAV hemagglutinin H1 lineage 1B (or human seasonal) circulating in the USA between January 2003 and October 2014. 507 sequences were included for this analysis. 25 sequences correspond to the sequences recovered during this study (VG2 and VG3 (n=15) and are color-coded by farm 1 (blue), 2 (aqua), 3 (magenta), 4 (orange), and 5 (red). Local support values for each node under the discrete gamma model with 20 rate categories (Gamma20-based likelihood) are illustrated based on the background color of the tree.

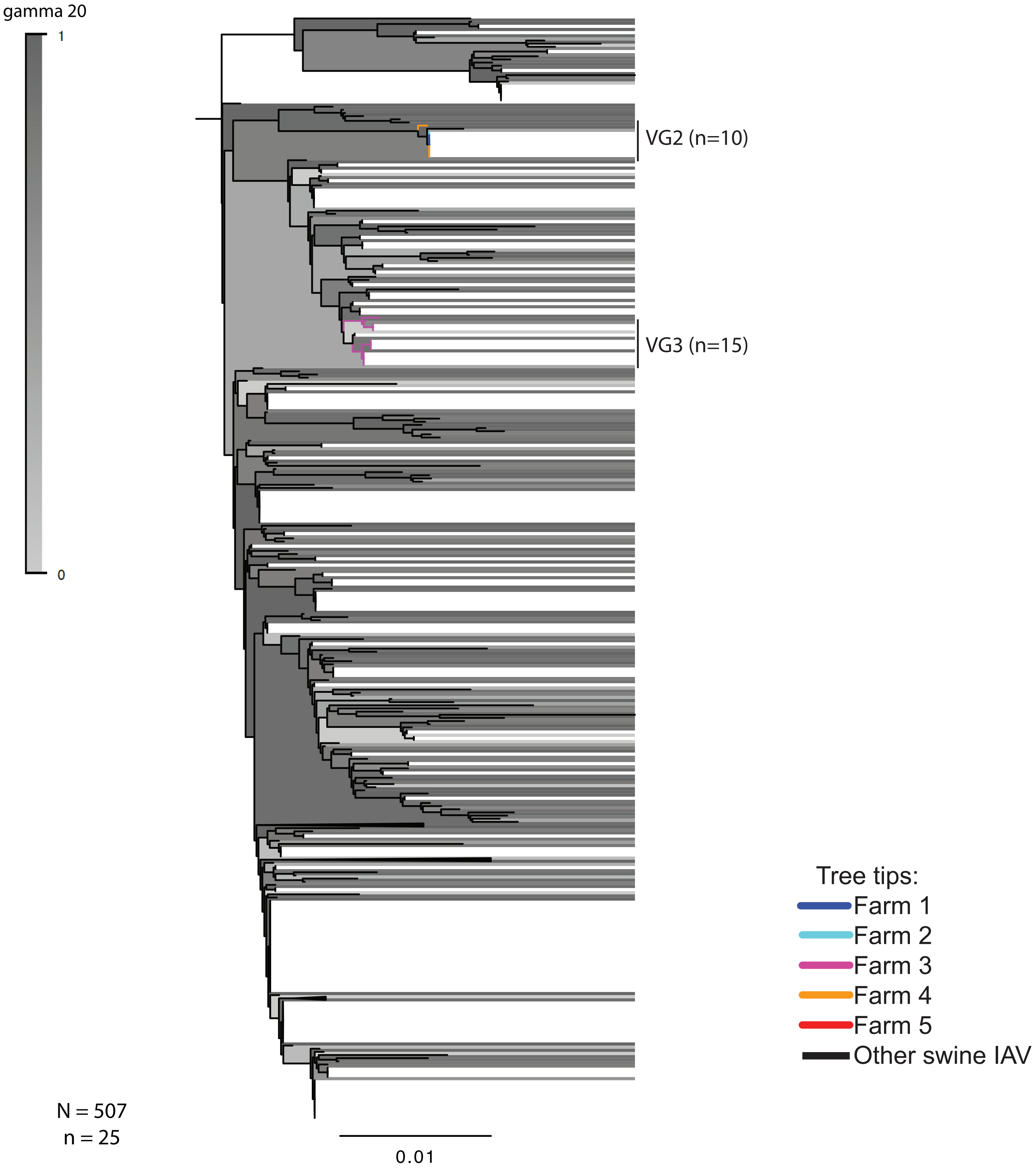

Supplemental material 3. Approximately maximum-likelihood tree for swine IAV hemagglutinin H3 circulating in the USA between January 2003 and October 2014. 838 sequences were included for this analysis. 67 sequences correspond to the sequences recovered during this study (VG4, VG5, VG6, and VG7) and are color-coded by farm 1 (blue), 2 (aqua), 3 (magenta), 4 (orange), and 5 (red). Local support values for each node under the discrete gamma model with 20 rate categories (Gamma20-based likelihood) are illustrated based on the background color of the tree.

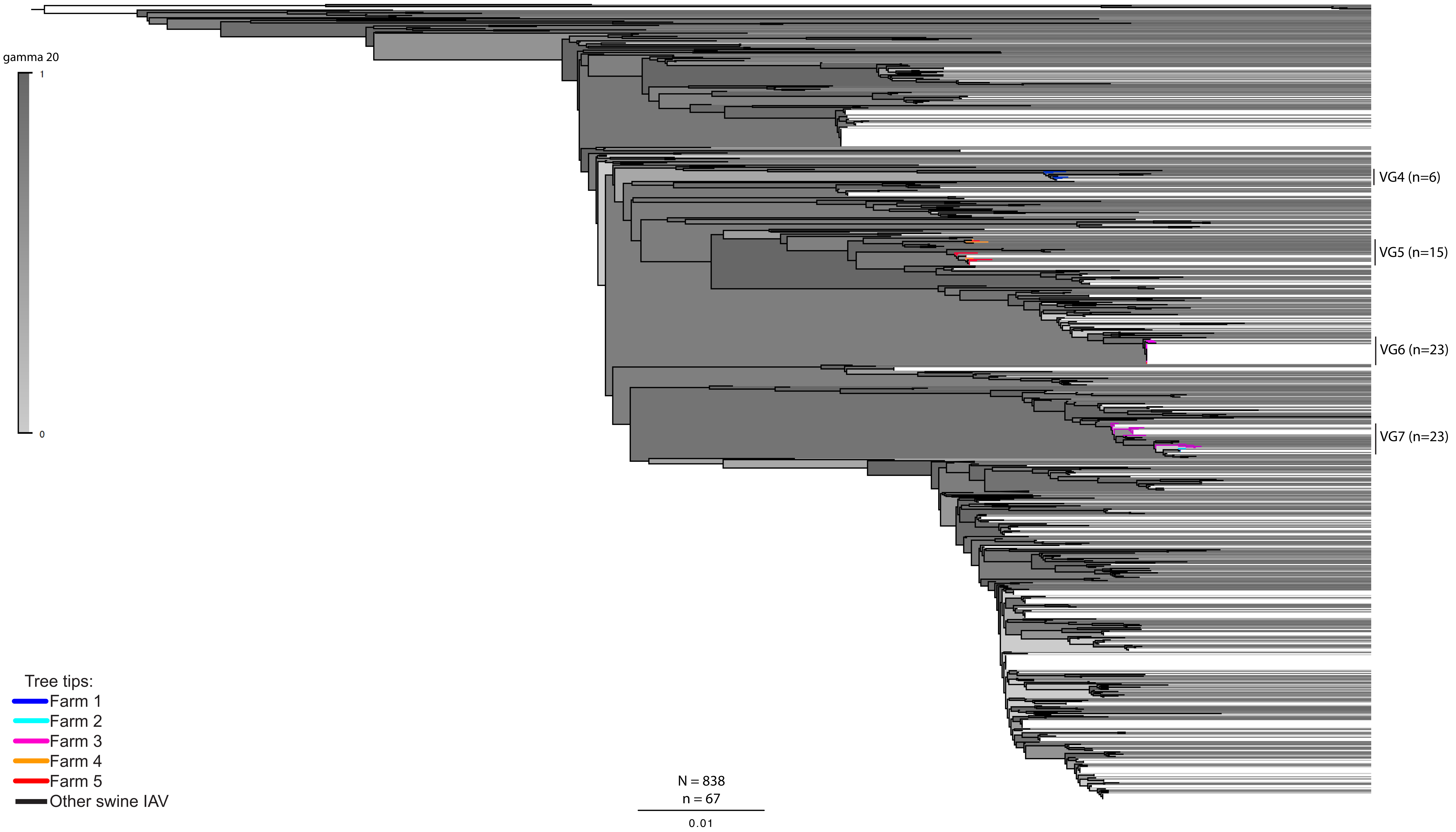

Supplement: Supplemental material [file JVI.00745-17_zjv999182878s1.pdf]
